# A Statistical Approach for Signal and Power Integrity Co-Design in High-Speed Interconnects Considering Non-Linear Power/Ground Noise and Bit-Patterns

**DOI:** 10.3390/mi14091654

**Published:** 2023-08-22

**Authors:** Youngwoo Kim

**Affiliations:** Department of Semiconductor System Engineering, Sejong University, Seoul 05006, Republic of Korea; youngwoo@sejong.ac.kr; Tel.: +82-2-3506-3408

**Keywords:** statistical approach, signal/power integrity, high-speed interconnects

## Abstract

In this article, a novel statistical approach is proposed and applied to co-design signal and power integrity (SI/PI) in high-speed interconnects considering the non-linear power/ground noise generated by parallel buffers and bit-patterns. With increased data rates and decreased operating voltages, the allowed noise margin in high-speed interconnects is continuously reduced, and this trend requires SI/PI co-design. Specifically, non-linear power/ground noise associated with simultaneous switching circuits sharing a power delivery network (PDN) and bit-patterns must be carefully considered during the interconnects’ design and analysis phase. In many cases, conventional electromagnetic (EM) and transient circuit simulators require heavy computational resources or even fail to deliver an accurate result. The proposed statistical method estimates the statistical eye-diagram in the high-speed interconnect considering power/ground noise and bit-patterns such as data bus inversion (DBI) coding. The accuracy and computational efficiency of the proposed method are validated by comparing the result with HSPICE transient simulation result. The proposed method is also compared with conventional statistical methods, such as peak distortion analysis (PDA) and statistical channel simulation in the transient simulator. Lastly, the proposed method is applied to the SI/PI co-design and co-analysis in the high bandwidth memory (HBM) interposer channel. Impacts of decoupling capacitors on hierarchical PDN impedance, statistical eye-diagram of the HBM channel, and bit error rate (BER) Bathtub curves are summarized. Finally, the BER eye-diagram is derived from the estimated statistical eye-diagram for timing and voltage analysis. The impacts of hierarchical PDN design and bit-patterns on SI/PI are discussed.

## 1. Introduction

Recent technologies driven by artificial intelligence, cloud-computing, and big-data, require terabyte/s (TB/s) bandwidth. Therefore, the data rate of DRAM and the number of parallel buffers have increased to support TB/s bandwidth. To support TB/s, data rates per pin in DRAM are continuously increasing regardless of DRAM types. Specifically, the data rate per pin in the Graphic DRAM (GDDR) for high-resolution image signal processing and artificial intelligence is increasing rapidly. High bandwidth memory (HBM), which is in the form of a 2.5-dimensional integrated circuit (2.5D-IC) based on through silicon via (TSV) and interposer technologies, is also key to realizing recent technologies [1,2,3,4,5,6,7]. It is expected that the data rate of the HBM channel will increase up to 12 gigabits per second (Gb/s). In order to maintain the TB/s system bandwidth, maintaining signal integrity and power integrity (SI/PI) in the high-speed interconnect is crucial.

**Figure 1:**
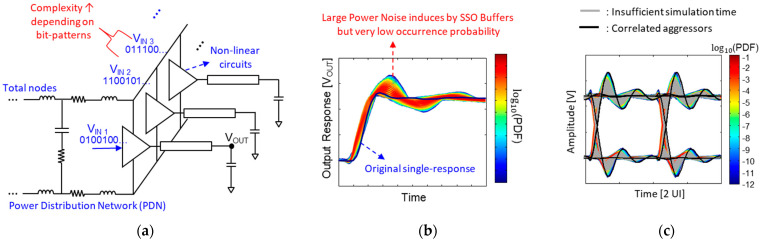
The limitation of conventional transient eye-diagram simulation/estimation methods is depicted. (**a**) Factors increasing complexity in transient simulations. (**b**) Power/ground noise due to SSO buffers sharing the PDN. (**c**) Discrepancy between an actual eye-diagram and simulated eye-diagram.

The high-speed channel suffers from various noise degrading SI, such as impedance mismatch, channel losses, cross-talks, and inter-symbol interference (ISI). Recently, due to the reduced noise margin associated with increasing data rates and decreasing operation voltages, noises related to PI must be considered together with SI. Switching of numerous logic circuits and input/output (I/O) buffers sharing the same power delivery network (PDN) generate simultaneous switching output (SSO) noise. Among non-linear power/ground noises, the power/ground noise induced by the SSO degrades the signal integrity of the high-speed interconnect severely and causes amplitude uncertainty in the output voltage of the transmitting or receiving buffers [8,9,10,11]. These noises are dominated by the occurrence probabilities of the buffer combinations, hierarchical PDN impedance, and bit patterns. To estimate a case that generates extremely large power/ground noise, large computational cost and time are required, since the occurrence probability of such a case is very low. An accurate eye-diagram simulation/estimation is extremely important, since it includes various SI/PI factors, and it is widely used to judge the SI/PI and electrical performance of the high-speed interconnect. In some cases, conventional transient simulators and eye-diagram estimation methods fail to accurately estimate the eye-diagram considering these noise effects, which is depicted in Figure 1. 

As the number of SSO buffers increases similarly to the HBM, large computational resources are needed to simulate accurate output responses and eye-diagrams of high-speed interconnect using conventional transient simulators. The input/output buffer information specification (IBIS) model is also known to be inaccurate under the impact of large power/ground noise. 

Statistical eye-diagram estimation methods based on single bit response (SBR) such as peak distortion analysis (PDA) enable fast and accurate SI analysis in the high-speed interconnect [12,13,14,15]. However, the SBR method is only applicable for the linear and time invariant (LTI) systems; therefore, the non-linear power/ground noise and PI factors cannot be considered for the SBR method. Double edge response (DER) and multiple edge response (MER) methods are capable of capturing the non-linearity impacts, but they are difficult to reflect the impacts of SSO noise [16,17,18,19]. In previous works [20,21], the statistical eye-diagram estimation method, which is capable of considering SSO impact, is proposed for the first time. However, the steady-states response affected by the SSO are not considered. When the number of parallel buffers increases, even though the victim buffer is in steady-state (digital state ‘zero’ or ‘one’), SSO buffers generate large amount of voltage fluctuations which affect not only eye-diagram contours but also overshoot/undershoot voltage characteristics. 

In this article, a novel statistical approach is proposed and applied to co-design signal and power integrity (SI/PI) in high-speed interconnects. Probability density functions (PDFs) of output step-responses and steady-state responses affected by the various noises are derived. Output responses are derived from repetitive step-response simulations using HSPICE. For each response, occurrence probability is calculated to derive the PDFs. In this step, it is possible to consider the bit pattern impacts mathematically. From the derived output response PDFs, main-cursor (MC) PDFs and inter-symbol interference (ISI) PDFs are defined based on the data rate. Statistical output responses are derived based on recursive convolution between the MC-PDFs and ISI-PDFs. Finally, a statistical eye-diagram is formulated by superposing the derived statistical output responses and expanding to two unit intervals (UIs). The accuracy and computational efficiency of the proposed method are validated by comparing the result with HSPICE transient simulation result. The proposed method is also compared with conventional statistical methods such as peak distortion analysis (PDA) and statistical channel simulation in the transient simulator. 

Lastly, the proposed method is applied to SI/PI co-design and co-analysis in the high bandwidth memory (HBM) interposer channel. The impacts of decoupling capacitors on hierarchical PDN impedance, statistical eye-diagram of the HBM channel, and bit error rate (BER) Bathtub curves are summarized. Finally, the BER eye-diagram is derived from the estimated statistical eye-diagram for timing and voltage analysis. The impacts of hierarchical PDN design and bit-patterns on SI/PI are discussed.

## 2. Statistical Eye-Diagram Estimation Method Considering Power/Ground Noise Due to SSO Buffers and Bit-Patterns

In this section, a new statistical method, which is capable of estimating an accurate statistical eye-diagram of the high-speed interconnect, is proposed. From the estimated statistical eye-diagram, various SI/PI indicators, such as BER bathtub curves and BER eye-diagrams, can be derived. In the derived/estimated results, the impacts of various power/ground noise, hierarchical PDN, channel factors, and bit-patterns are included. 

### 2.1. Step Responses Derivation

**Figure 2:**
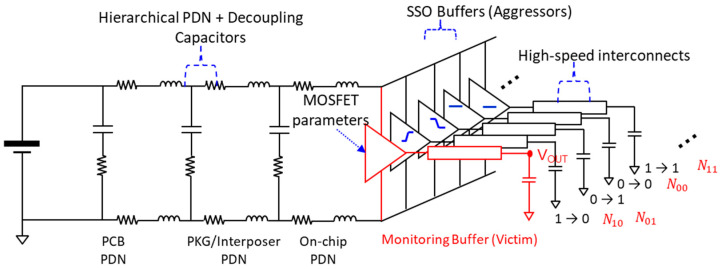
Considerable parameters for deriving output responses considering non-linear power/ground noise and SSO effects. Each state is defined as $N_{01}$, $N_{10}$, $N_{00}$, and $N_{11}$ in this research and will be used as variables to derive output responses and occurrence probabilities (data coding/bit-pattern impacts).

Similar to other statistical methods, output responses and occurrence probability of the responses must be derived. In this subsection, a method to derive the output responses is explained. To derive output responses, design parameters affecting output responses and variables should be defined. In Figure 2, an equivalent circuit model with considerable parameters for deriving output responses are shown. The hierarchical power distribution network (PDN), which consists of on-chip PDN, package/interposer PDN, printed circuit board (PCB) PDN, and decoupling capacitors in each PDN, must be considered. To accurately reflect the ground noise, the PDN must be decomposed into the power and ground interconnects instead of the S-parameter model. If the PDN is modeled based on the S-parameter model, such as the touchstone file, ground noise will be added to the power noise, which results in exaggerated power noise and suppressed ground noise [22,23,24]. However, high-speed interconnects can be modeled using S-parameter models or equivalent circuit models. These parameters should be carefully chosen or extracted depending on a target system to be analyzed, since these parameters determine overall output response characteristics.

This equivalent circuit can be mathematically modeled into simultaneous second order differential equations [20]. In such case, the final eye-diagram can be estimated solely using a mathematic tool such as MATLAB. However, mathematical modeling itself requires an insight, and it is also time consuming. In this work, the transient simulator HSPICE is used for the equivalent circuit. Still, only responses will be derived, so it requires a much smaller computational resource and time compared to a full eye-diagram simulation. The output response characteristics and occurrence are determined by the SSO buffer states. Agressor buffers can be in four different states. Therefore, the four different states of aggressor SSO buffer are set as variable to derive output responses and occurrence probabilities. The SSO combinations are determined by the four variables, which are the number of SSO buffers in pull-up/down transitions ($N_{01}$/$N_{10}$) and the number of SSO buffers remaining in steady states logic ‘zero’ ($N_{00}$), and logic ‘one’ ($N_{11}$), as shown in Figure 2 with red letters. If these buffers are randomly operating (un-correlated to each other), it is not coded. If the occurrence probability of each buffer is correlated somehow, it is coded and in this case, bit patterns must be considered. In the following subsection, the occurrence probabilities of the SSO buffers for random cases and coded cases are derived.

**Figure 3:**
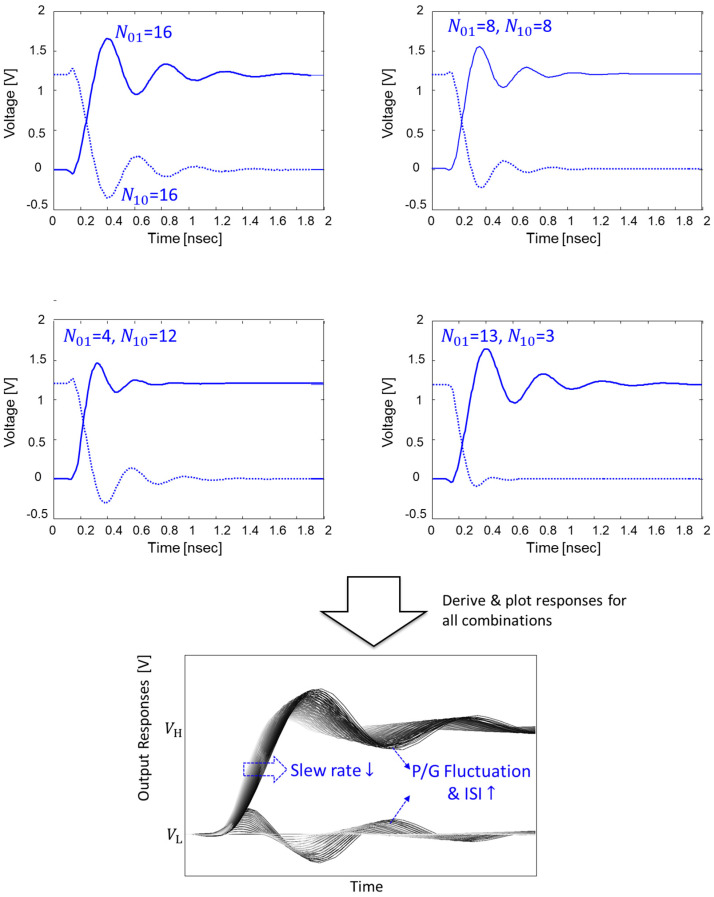
Output responses for specific buffer combinations are shown. All responses are derived and plotted. Power/ground noise due to SSO buffers degrades slew-rate and causes voltage uncertainty.

For step-input response, ‘one-to-zero’ for pull-up, ‘zero-to-one’ for pull-down, ‘zero-to-zero’ for steady state ‘one’, and ‘one-to-one’ for steady state ‘zero’ are injected. For each step-input response to the victim buffer, the state of the SSO buffers are varied. If the total number of buffers is set as $N_{T}$, there are $4^{\left(N_{T}-1\right)}$ SSO combinations for each step-response. However, the SSO buffers remaining in steady states have minimal impacts on the output responses of the victim buffer. Therefore, output responses for cases when $N_{01}+N_{10}\leq N_{T}-1$ are considered for more efficient derivation. By considering the above condition, a considerable SSO combination number reduces from $4^{\left(N_{T}-1\right)}$ to $N_{T}(N_{T}+1)/2$. In Figure 3, derivation of output responses is graphically explained. For all cases, step responses are derived and plotted. As can be seen from Figure 3, if all buffers are in the same pull-up or pull-down transition, response with a large fluctuation and slower slew-rate is derived due to large power/ground noise and SSO noise. With the same number of pull-up and pull-down buffers, power and ground currents are canceled out, resulting in reduced fluctuation with a faster slew-rate. The number of pull-up or pull-down transitions increases, and larger power/ground noise with slower slew-rate are determined. Occurrence probability of each output response should be considered to derive a statistical eye-diagram.

### 2.2. Occurrence Probability Calculation

To formulate the output responses to the PDF form, the occurrence probability of each output response should be determined. Depending on bit patterns and data-coding properties, the probability calculation can be different. In this subsection, two cases are given as representative; a case when buffers sharing the same PDN are uncorrelated to each other, and a case when the data bus inversion (DBI) coding is adopted to reduce the power/ground noise and crosstalk. 

#### 2.2.1. Uncorrelated Buffers (Random)

To derive output response PDFs, the occurrence probability of each SSO combination should be derived. The occurrence probability of SSO can be calculated as in Equation (1), where $N_{SSO}$ is the total number of aggressor SSO buffers, $N_{T}-1$, excluding the number of victim buffers

(1) $$P_{m}\left(N_{01},N_{10},\;N_{11},N_{00}\right)={\left(\frac{1}{2}\right)}^{2}\times {\left(\frac{1}{4}\right)}^{N_{SSO}}\times_{N_{SSO}}C_{N_{00}}\times_{\left(N_{SSO}-N_{00}\right)}C_{N_{01}}\times_{\left(N_{SSO}-N_{00}-N_{01}\right)}C_{N_{10}},\;m=\;00,\;11,\;01,\;\&\;10$$

In (1), $\frac{1}{2}$ is the transition probability of the victim buffer and $\frac{1}{4}$ is the transition probability of each SSO buffer. Since the number of SSO buffers remaining in steady states, $N_{11}$ and $N_{00}$, has negligible impact on the victim buffer, (1) can be rewritten by adding up the probabilities of $N_{11}$ or $N_{00}$, which is summarized in Equation (2)

(2) $$P_{m}(N_{01},N_{10})\;\;=\;\;\sum_{N_{00}\;(orN_{11})=0}^{N_{SSO}-N_{01}-N_{10}}{\;\;P}_{m}(N_{01},N_{10},N_{11},N_{00})$$

**Figure 4:**
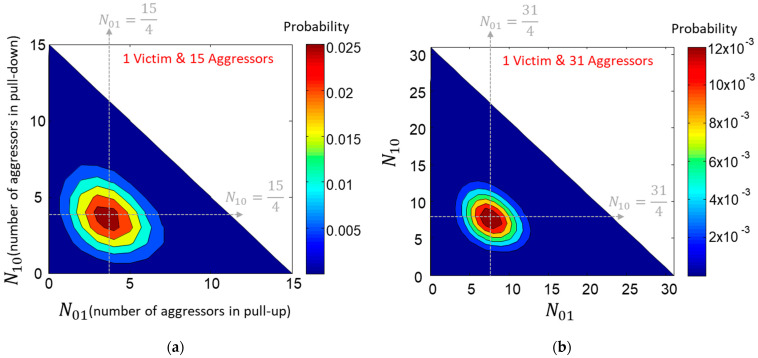
Occurrence probabilities for $N_{T}=16$ are plotted in (**a**). Occurrence probabilities for $N_{T}=32$ are plotted in (**b**). For both cases, the probability around $N_{01}=N_{10}={(N}_{T}-1)/4$ is the largest.

In Figure 4, the occurrence probability distributions are given as examples by calculating and plotting the occurrence probability of each response for a case when $N_{T}=16$ (Figure 4a) and a case when $N_{T}=32$ (Figure 4b). The probability is highest when both $N_{01}$ and $N_{10}$ are near $\frac{{(N}_{T}-1)}{4}$. Unless the SSO buffers are coded, cases when all SSO buffers are evenly distributed are the most likely to appear.

#### 2.2.2. Data Bus Inversion (DBI) Applied

When buffers are correlated to each other (data coding), the impacts of data coding can be reflected by calculating the occurrence probability of each coding. In this sub-section, DBI-coding is given as an example, since this coding has been widely adopted in memories to suppress power/ground noise and crosstalk as a standard. 

When DBI coding is applied, the case when $N_{01}+N_{10}>N_{T}/2-1$ should be eliminated by the bit inversion. In this case, occurrence probability of $P_{00\;and\;11}(N_{01},N_{10})$ and $P_{01\;and\;10}(N_{01},N_{10})$ must be separately calculated, since the latter one, the victim buffer, is already making a bit transition. When the DBI coding is occurred, $P_{m}(N_{01},N_{10},N_{00},N_{11})$ becomes $P_{m-complimentary}(N_{00},N_{11},N_{01},N_{10})$ and should be added up to the corresponding occurrence probability when DBI coding has not occurred. In Table 1, occurrence probabilities are calculated and summarized.

**Figure 5:**
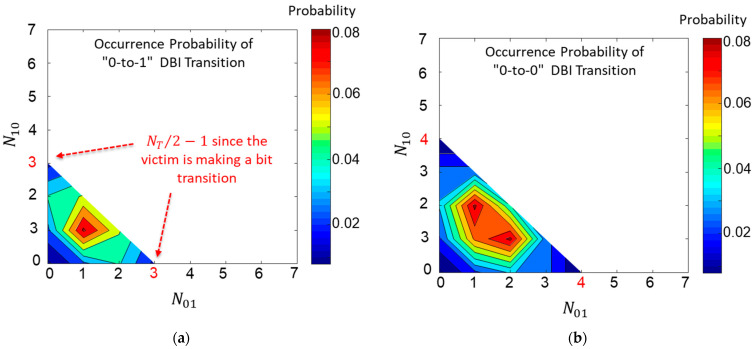
Occurrence probabilities for the case $N_{T}=8$ with DBI coding are plotted. (**a**) $P_{01\;(or\;10)-DBI}(N_{01},N_{10})$. (**b**) $P_{00\;(or\;11)-DBI}(N_{01},N_{10})$.

In Figure 5, the occurrence probabilities are plotted for the case $N_{T}=8$ with DBI coding. When data coding is applied, $P_{01\;(or\;10)-DBI}(N_{01},N_{10})$ and $P_{00\;(or\;11)-DBI}(N_{01},N_{10})$ must be different. For the case without data coding, $P_{01\;(or\;10)}(N_{01},N_{10})$ and $P_{00\;(or\;11)}(N_{01},N_{10})$ should be equal. When all occurrence probabilities are summed up, for the case without data coding, both $\sum P_{01\;(or\;10)}(N_{01},N_{10})$ and $\sum P_{00\;(or\;11)}(N_{01},N_{10})$ should also be equal to $\frac{1}{2}$. This is natural, since each pattern is occurring randomly. However, when the data coding is applied, it has now changed. In case of the DBI coding, buffers remaining in the steady states have higher occurrence probabilities ($\sum P_{01\;(or\;10)}(N_{01},N_{10})$ < $\sum P_{00\;(or\;11)}(N_{01},N_{10})$), since the purpose of the DBI coding is to suppress the aggressor SSO buffer transitions below $N_{01}+N_{10}<N_{T}/2$.

### 2.3. Statistical Output Responses (SORs) Derivation Based on Recursive Convolution

**Figure 6:**
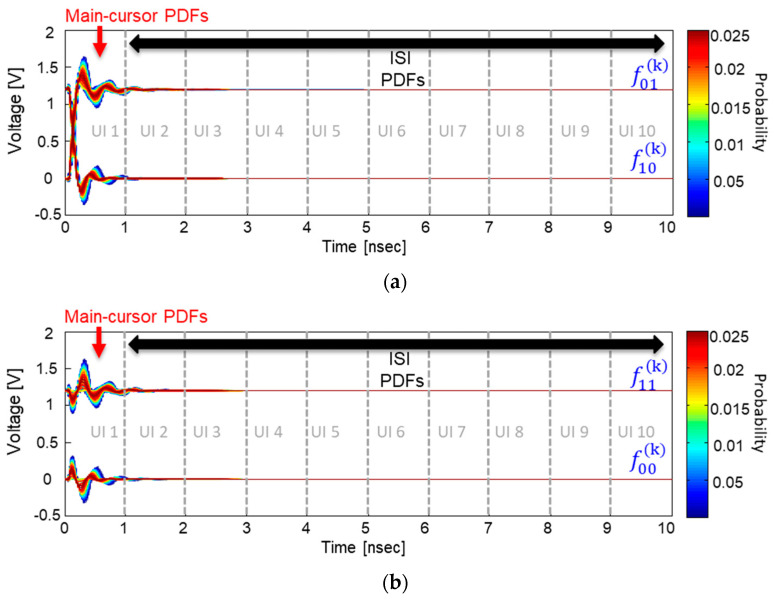
Derived output responses are plotted. Steady-states (**a**) and pull-up/down responses (**b**) are shown, respectively. In the main-cursor PDFs, voltage uncertainty is clearly shown. In the ISI PDFs, various noises impacts are included.

To derive a statistical eye-diagram, including various noises affecting SI/PI, we must define main cursor-PDFs and ISI PDFs. In Figure 6, voltage variation PDFs extracted from the pull-up output response PDF are relocated to the quantized voltage-time planes. In this paper, the main cursor PDF matrixes are defined as Equation (3), and the ISI PDF matrixes are defined as Equation (4). In Figure 6, the main-cursor PDFs and ISI PDFs are also marked.

(3) $$f_{m}^{\left(1\right)},\;\mathrm{where}\;m=01,10,00,\&\;11$$

(4) $$f_{m}^{\left(k\right)},\;\;\mathrm{where}\;m=01,10,00,\&\;11\;and\;k=2,3,\ldots ,\infty$$

The responses remaining at the main cursors generating distortions are regarded as an ISI. The ISI can be generated by not only pull-up or pull-down transitions but also steady-state output responses fluctuated by the SSO buffers. Without the SSO buffers, the PDFs of an individual ISI can be represented as the Kronecker delta functions. However, due to voltage uncertainties generated by the SSO buffers, the Kronecker delta functions should be replaced by the ISI-PDFs defined in (4). The total ISI-PDFs, including the various noises, can be calculated by a recursive convolution between ISI-PDFs affecting each other along the voltage axis at every sample time. Results of recursive convolution between ISI-PDFs at the $k^{th}$ previous unit intervals (UIs) are summarized in Equations (5)–(8). In the (5)–(8), ${{ISI}_{m}}^{-\left(k\right)}\left(t,v\right)$, where m=00, 11, 01, and 10, is ISI-PDF at the $k^{th}$ previous UIs, which can be calculated by taking the convolution between the self-generated ISI, ${f_{m}}^{\left(k+1\right)}$ and ISI-PDFs until the ${\left(k+1\right)}^{th}$ previous UIs, ${{ISI}_{m}}^{-\left(k+1\right)}\left(t,v\right)$. In this article, ISI-PDFs are defined as (4), where the k is counted from 2. Therefore, the self-generated ISIs at the $k^{th}$ previous UIs are defined as ${f_{m}}^{\left(k+1\right)}$, not the ${f_{m}}^{\left(k\right)}$. In (5)–(8), new mathematical operators ${Shift}_{0}$ and ${Shift}_{1}$ are adopted, which shifts the ISI-PDFs to the logic state “zero” or “one,” since it is important to redefine the location of the ISI-PDFs to the true voltage level of logic “one ($V_{H}$)” and “zero ($V_{L}$)” after the convolution [16]. For example, ISI-PDFs in the logic state “zero” should remain in the logic “zero” state, which is $V_{L}$. Therefore, in (5) and (7), ${Shift}_{0}$ operator is used to relocate the PDFs to the $V_{L}$. The ISI-PDFs in the logic “one” should remain in the logic “one” state, which is $V_{H}$. Therefore, in (6) and (8), ${Shift}_{1}$ operator is used to relocate the PDFs to the $V_{H}$. The initial conditions for the ISI-PDFs at the k=∞ are defined by the Kreonecker delta functions summarized in (9). For an efficient recursive convolution, each PDF is scaled four times. Thus, at the final step, when deriving the statistical responses, 14 should be multiplied.

(5) $$\begin{array}{c} {{ISI}_{00}}^{-(k)}\left(t,v\right)=\sum_{m=00}^{01,10,11}{Shift}_{0}\left(\frac{{f_{00}}^{\left(k+1\right)}}{4}⊗{{ISI}_{m}}^{-\left(k+1\right)}\left(t,v\right)\right) \end{array}$$

(6) $$\begin{array}{c} \;{{ISI}_{01}}^{-(k)}\left(t,v\right)=\sum_{m=01}^{10,11,00}{Shift}_{1}\left(\frac{{f_{01}}^{\left(k+1\right)}}{4}⊗{{ISI}_{m}}^{-\left(k+1\right)}\left(t,v\right)\right) \end{array}$$

(7) $$\begin{array}{c} {{ISI}_{10}}^{-(k)}\left(t,v\right)=\sum_{m=10}^{11,00,01}{Shift}_{0}\left(\frac{{f_{10}}^{\left(k+1\right)}}{4}⊗{{ISI}_{m}}^{-\left(k+1\right)}\left(t,v\right)\right) \end{array}$$

(8) $$\begin{array}{c} {{ISI}_{11}}^{-(k)}\left(t,v\right)=\sum_{m=11}^{00,01,10\;}{Shift}_{1}\left(\frac{{f_{11}}^{\left(k+1\right)}}{4}⊗{{ISI}_{m}}^{-\left(k+1\right)}\left(t,v\right)\right) \end{array}$$

(9) $${{ISI}_{10,00}}^{-(\infty )}\left(t,v\right)=\delta \left(v-V_{L}\right)\;\&\;{{ISI}_{01,11}}^{-(\infty )}\left(t,v\right)=\delta \left(v-V_{H}\right)$$

Statistical output responses (SORs) affected by the ISI-PDFs can be derived by taking the final convolution between main cursor-PDFs and ISI-PDFs affecting each other. The SOR can be derived by calculating (5)–(8) at k=0. Finally, half (1 UI) part of a statistical eye-diagram can be obtained using Equation (10)

(10) $$Stateye_{1UI}=\frac{1}{4}\;({{ISI}_{00}}^{-(0)}\left(t,v\right)+{{ISI}_{11}}^{-(0)}\left(t,v\right)+{{ISI}_{01}}^{-(0)}\left(t,v\right)+{{ISI}_{10}}^{-(0)}\left(t,v\right))$$

**Figure 7:**
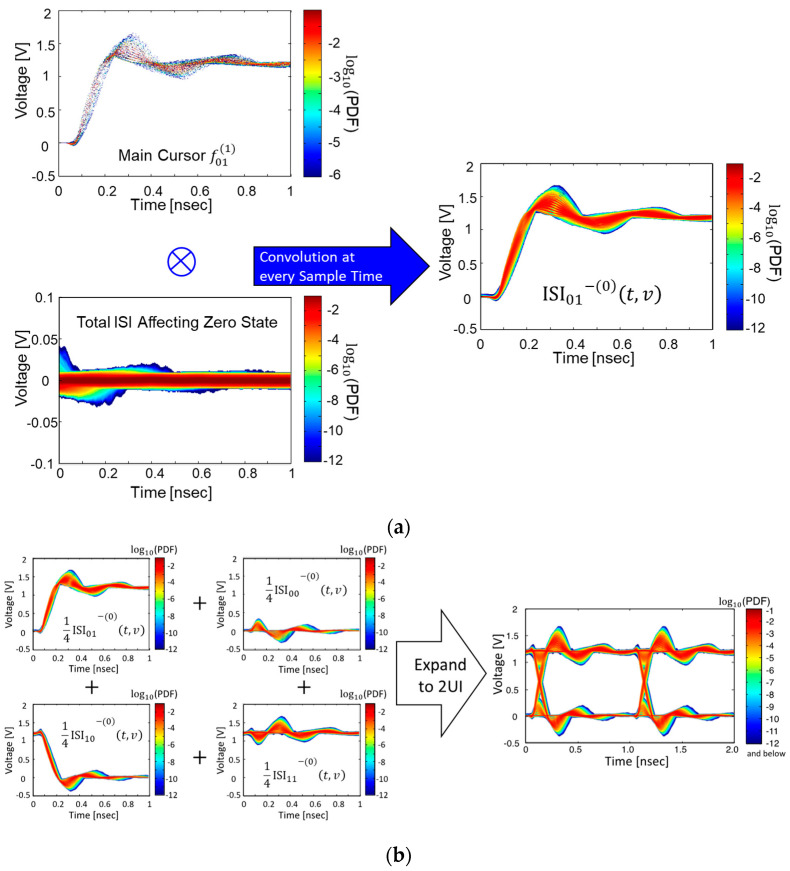
Derivation of statistical output responses (SORs) are graphically depicted. (**a**) Final convolution to derive ${{ISI}_{01}}^{-(0)}\left(t,v\right)$ is shown. It is a convolution between main cursor PDF ${f_{01}}^{\left(1\right)}$ and total ISI affecting the logic zero state. (**b**) Derivation process of the final eye-diagram is shown.

If (10) is expanded to 2 UIs, the final statistical eye-diagram, including various noises affecting the SI/PI, can be derived. In Figure 7, the proposed procedure is graphically depicted. From the derived statistical eye-diagram, BER can be derived by considering the random jitter and then calculating BER at every sample timing and voltage. The random jitter can be considered by taking a convolution between the derived statistical eye-diagram and random jitter distribution at every voltage level. 

In the following sections, the proposed method is verified and applied for the SI/PI co-design and co-analysis for the high-speed interconnect. 

## 3. Verification of the Proposed Statistical Method

The proposed method is verified in this section. First, the proposed method is directly compared with HSPICE transient simulation for cases with small number of SSO buffers. In this case, the occurrence probabilities of some dramatic cases such as all aggressors in the same transitioning state are not extremely low. This means that those cases are likely to appear during the transient simulation. In these cases, accuracy of the proposed method can be directly compared with full transient simulation. Then, the proposed method is compared to more complex cases. Lastly, the proposed method is compared with previous works to emphasize the accuracy. 

**Figure 8:**
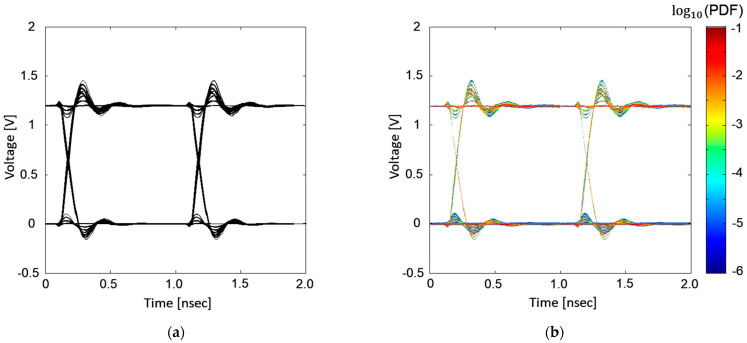
Total of four buffers operating at a data rate of 1 Gb/s. (**a**) HSPICE simulation result. (**b**) Estimated statistical eye-diagram using the proposed method.

In Figure 8, case for four buffers sharing the same hierarchical PDN with a data rate of 1 Gb/s is verified by comparing the estimated statistical eye-diagram with the transient eye-diagram simulated using HSPICE. Since the lowest occurrence probability of worst-cases (all aggressor buffers in pull-up or pull-down transition) is around 0.01, which is likely appear in the transient simulation, direct comparison between two eye-diagrams is possible. In case with four buffers, the estimated eye-diagram showed good correlation with that obtained from the direct transient simulation. However, as shown in Figure 8a, the voltage variations which have occurrence PDFs below $10^{-4}$ order, the transient simulation starts to lose accuracy or requires longer simulation time. To mitigate this issue in the transient simulation, simulation with longer bit patterns and longer simulation time should be conducted.

**Figure 9:**
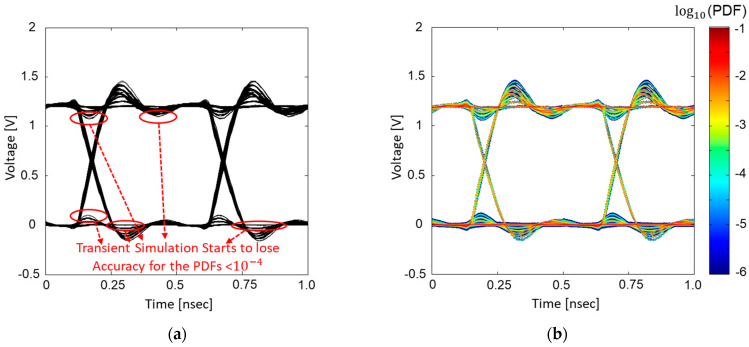
Total of four buffers operating at a data rate of 2 Gb/s. (**a**) HSPICE simulation result. (**b**) Estimated statistical eye-diagram using the proposed method.

In Figure 9, the case for the four buffers with a data rate of 2 Gb/s is verified by comparing the estimated statistical eye-diagram with transient eye-diagram simulated in HSPICE. With a higher data rate, the eye-opening voltage is decreased and timing jitter is increased, since more UIs are considered in the recursive convolution when defining the ISI-PDFs. In this case, transient simulation starts to lose accuracy with voltage variations, with occurrence probability below $10^{-4}$ order. If the number of aggressor buffers increase, allowed aggressor combinations will dramatically increase with lower occurrence probabilities. Therefore, if the number of the aggressor buffer increases, transient simulation will lose accuracy or even fail to estimate some responses or noise characteristics. 

In Table 2, accuracy and simulation time (estimation time for the proposed method) comparisons between the HSPICE transient simulation and the proposed method are summarized. Conventional transient simulation is limited when the number of buffers increases. As the number of buffers increases, the transient simulation loses its accuracy to achieve precise eye-diagrams. Recently, BER analysis has become even more important, and this analysis begins from the obtained statistical eye-diagram. The BER obtained using the transient simulation is losing accuracy below $10^{-4}$ order. The proposed method is capable of estimating BER below $10^{-16}$ order or even lower with a higher data rate.

**Figure 10:**
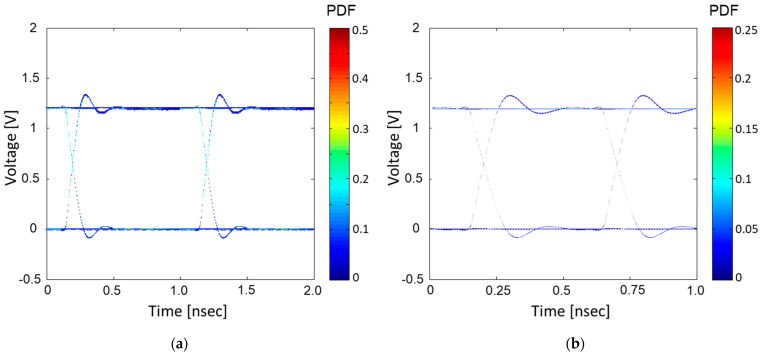
Estimated eye-diagrams based on the PDA method are plotted. (**a**) four buffers operating at 1 Gb/s and (**b**) four buffers operating at 2 Gb/s. Compared to Figure 8 and Figure 9, the PDA method is not capable of estimating an accurate eye-diagram under the influence of power/ground noise.

The proposed method is compared with previous works. First, the proposed method is compared with the peak distortion analysis (PDA) method [12]. In Figure 10, estimated eye-diagrams obtained using [12] are plotted for the comparison. For both Figure 10a,b, four buffers sharing the hierarchical PDN are set, but they operate at different data rates: 1 Gb/s and 2 Gb/s. Compared to Figure 8 and Figure 9, the PDA method is not capable of estimating an accurate eye-diagram under the influence of power/ground noise.

**Figure 11:**
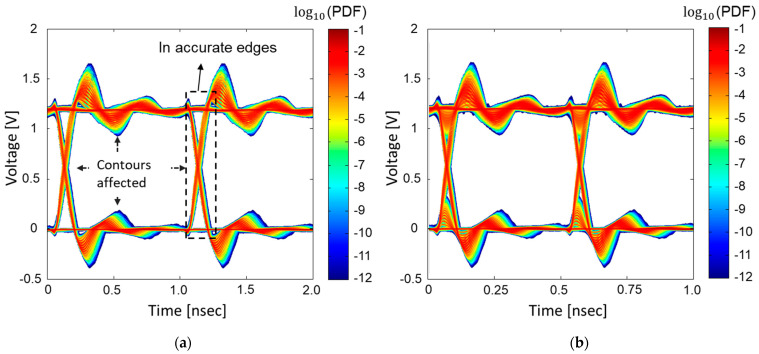
Comparison between statistical eye-diagrams estimated based on previous methods [20,21] (**a**) and estimated based on the proposed method. (**b**) Total of 16 buffers are in operation, generating SSO effects at the data rate of 1 Gb/s.

Previous works [20,21] proposed the statistical eye-diagram estimation method considering power/ground noise generated by SSO for the first time. However, it does not consider steady states affected by SSO. In Figure 11, statistical eye-diagrams estimated based on previous methods [20,21] and the proposed method are compared. Without consideration of the steady states affected by SSO, estimated statistical eye-diagrams have inaccurate contours, rising/falling edges, and steady states. 

In the following section, the proposed method is used to SI/PI design in the high-speed interconnect. 

## 4. SI/PI Co-Design in the High-Speed Interconnect Using the Proposed Method

In this section, the proposed method is applied for SI/PI co-design in the high-speed interconnect, targeting an HBM interposer channel. HBMs are assembled on a silicon interposer to form a 2.5-dimensional system for high system performance with a small form factor. The silicon interposer plays an important role in TB/sec bandwidth, as in the interposer, ultra-fine signal, power, and ground interconnects can be designed and fabricated in small dimensions. Due to complex design and fine dimensions, the PDN of the interposer is resistive and inductive, resulting in a high hierarchical PDN impedance. The PDN impedance affects power/ground noise property, resulting in SI/PI degradation. At the early design stage, each design iteration requires SI/PI analysis. Thus, an efficient analysis method is extremely important these days. 

**Figure 12:**
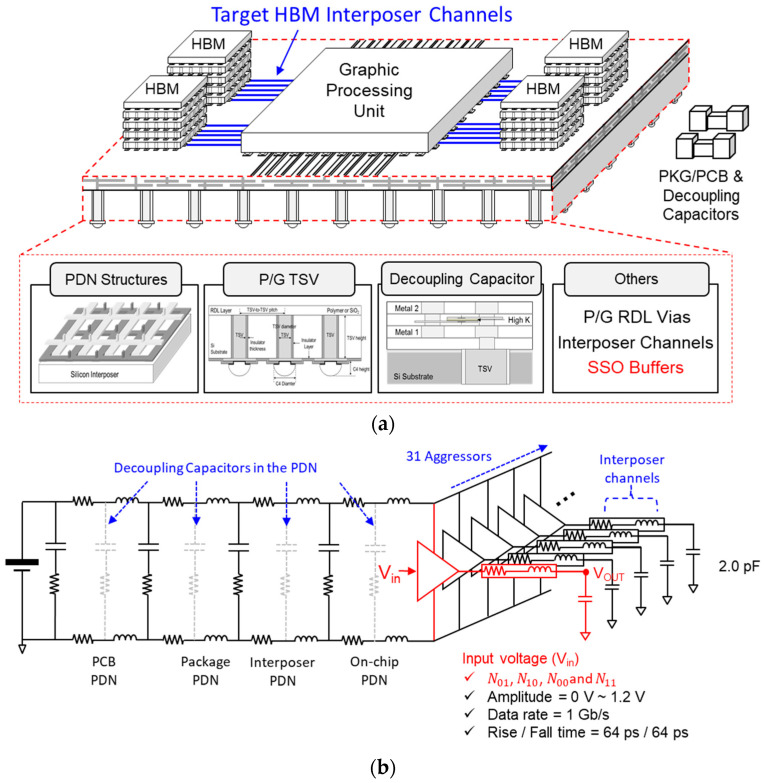
(**a**) Conceptual image describing a target high-speed channel to be designed and analyzed considering the parameters affecting the SI/PI of the high-speed channel. (**b**) An equivalent circuit model of target hierarchical PDN and buffers in the HBM interposer.

In Figure 12a, a conceptual image describing a target high-speed channel to be designed and analyzed is shown. Various design parameters affecting the SI/PI are depicted. In Figure 12b, an equivalent circuit model of the hierarchical PDN and buffers, which generate power/ground noise, is shown. In one sub-block of the HBM, there exist 32 buffers, therefore, 31 aggressors generate SSO noise and power/ground noise. As can be seen from the HBM ball map [1,2,3], power/ground interconnects in chip and interposer PDNs shield most of the power/ground noise coupling from adjacent sub-blocks. For the hierarchical PDN shown in the Figure 12a,b, actual PDN designs of HBM interposer, package, PCB and chip are considered based on reference [1] and related previous works [2,3,5,24,25]. However, if the PDN is modeled based on the S-parameter matrix extracted from electromagnetic (EM) simulator, ground interconnect will be added up to the power interconnect, this will result in inaccurate ground noise. Therefore, power/ground resistance and inductance are extracted from the hierarchical PDN impedance.

**Figure 13:**
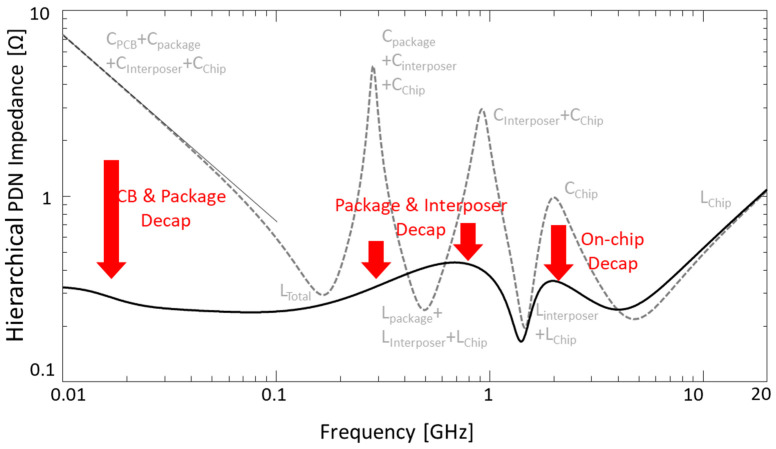
Hierarchical PDN impedances seen from the HBM are plotted without and with decoupling capacitor schemes. In the hierarchical PDN, on-chip, interposer, package and PCB PDNs are considered with decoupling capacitors in each PDN.

In Figure 13, hierarchical PDN impedance seen from the HBM is shown. As shown in Figure 12b, on-chip, interposer, package, and PCB PDNs are considered with decoupling capacitors in each PDN. Due to high inductance and resistance of the package and interposer PDN, hierarchical PDN impedance remains high at most of the frequency ranges with anti-resonance peaks. Proper decoupling capacitors should be placed in the PDN to reduce the hierarchical PDN impedance to solve SI/PI problems. 

When a proper decoupling capacitor scheme is adopted in the PDN, hierarchical PDN impedance is lowered in most of the frequency ranges with anti-resonance peaks removed. With a frequency range over 5 GHz, the impedance profile remains almost the same, even though decoupling capacitors are included. This can be solved by placing more on-chip decoupling capacitors in the on-chip PDN with smaller values. However, since the data rate of buffers in this case is 1~2 Gb/s, which is 0.5~1 GHz, current hierarchical PDN design is sufficient. 

**Figure 14:**
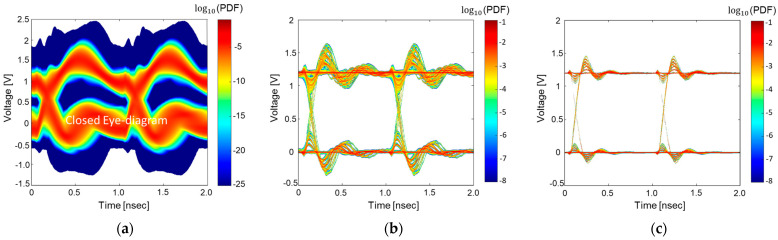
Estimate statistical eye-diagrams of the HBM interposer channel based on the proposed method. (**a**) Case without proper decoupling capacitor scheme in the hierarchical PDN. (**b**) With decoupling capacitor scheme. (**c**) When the DBI coding is adopted.

In Figure 14, estimate statistical eye-diagrams of the target HBM interposer channel based on proposed method are shown and compared. As can be seen from Figure 14a, without a proper decoupling capacitor scheme in the hierarchical PDN, increased power/ground noise due to the SSO buffers associated with high PDN impedance severely degrades the SI of the HBM interposer channel. It is apparent that designs related to PI must be considered simultaneously with SI analysis. The PDN design and optimal decoupling capacitor schemes affect both SI/PI of the high-speed interconnect. 

Figure 14b shows the changes in the statistical eye-diagram when the proper decoupling capacitor scheme is adopted in the hierarchical PDN. As shown in Figure 13, reduced hierarchical PDN impedance in wide frequency bands also suppressed power/ground noise, resulting in an improved eye-diagram. Figure 14c shows further improvement in the eye-diagram by adopting the DBI coding. When the buffers are coded, cases generating large power/ground noise and crosstalk are eliminated. Compared to Figure 14b, the eye-diagram is further improved in Figure 14c. By adopting the proposed method, impacts of various factors affecting SI/PI on the target high-speed interconnect are clearly visible. It is shown that the hierarchical PDN impedance improvement also ensured SI of the target high-speed interconnect. By adopting the coding, SI is further improved and impacts are clear.

The BER eye-diagram can be obtained by calculating the BER at every receiver sampling time and reference voltage offset as shown in Equation (11), where the $b_{m}$ represents the correct digital state, ‘1’ or ‘0’.

(11) $$BER\left(V_{ref}\right)=\frac{1}{2}P\left(V_{out}<V_{ref}\right|b_{m}=1)+\frac{1}{2}P\left(V_{out}>V_{ref}\right|b_{m}=0)\;$$

**Figure 15:**
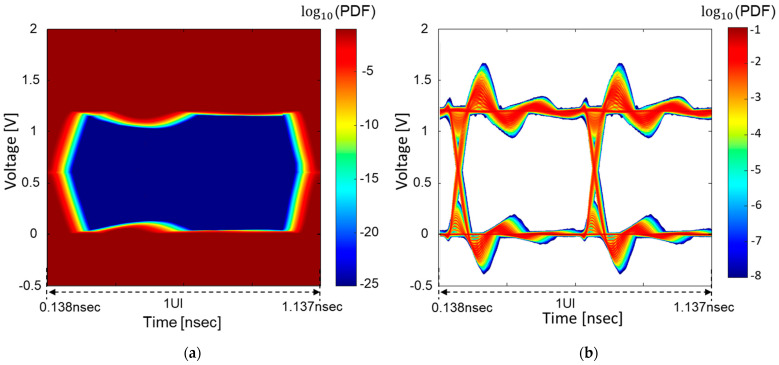
(**a**) The BER eye-diagram of the HBM channel is derived from the estimated statistical eye-diagram shown in Figure 14b. (**b**) The BER eye-diagram of the HBM channel is derived from the estimated statistical eye-diagram shown in Figure 14c. Note that scales are different.

**Figure 16:**
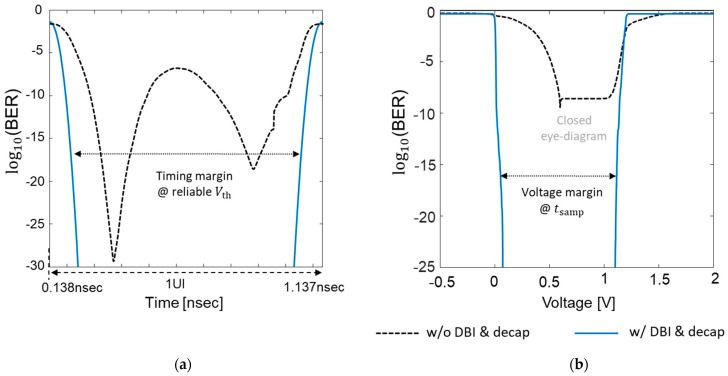
(**a**) Timing bathtub curves are compared at the threshold voltage ($V_{th}$). (**b**) Bathtub curves comparison at the sampling time.

In Figure 15, derived BER eye-diagrams are compared without and with DBI coding. By applying the DBI coding, the opening of the BER eye-diagram is increased. Note that the BER scale is different in Figure 15a,b. To derive the BER, (11) is calculated at every quantized sample voltage and time. Lastly, the timing and voltage bathtub curves are obtained from the derived BER eye-diagrams. The timing bathtub curves are compared at the threshold voltage ($V_{th}$) in Figure 16a. In Figure 16b, the voltage bathtub curves at the sampling time are plotted and compared. The impacts of the hierarchical PDN impedance and data coding on both the timing and voltage bathtub curves are clearly shown in Figure 16. Proper SI/PI co-design ensures reliable timing and voltage analysis as well.

By applying the proposed method, the target HBM channel affected by various noise factors can be easily and efficiently derived. Simultaneous comparison of the hierarchical PDN impedance and eye-diagram of the target interconnect is possible. From the derived statistical eye-diagram, further indicators such as BER eye-diagrams and timing/voltage bathtub curves can be formulated for an SI/PI co-design and co-analysis. 

## 5. Conclusions

In this article, a novel statistical approach, which is capable of reflecting various noises affecting the SI/PI of the high-speed channel, was proposed and verified. Probability density functions (PDFs) of output step-responses and steady-state responses affected by the various noises were derived using HSPICE. Output responses were derived from repetitive step-response simulations using HSPICE. Occurrence probability of each output response was calculated to formulate the PDFs. During this step, it is possible to consider the bit pattern and data-coding impacts mathematically. In this article, uncorrelated cases (randomly switching) and DBI coding were considered as representatives. From the derived output response PDFs, main-cursor (MC) PDFs and inter-symbol interference (ISI) PDFs were defined. After recursive convolution between the MC-PDFs and ISI-PDFs, four final statistical output responses are derived. Finally, the statistical eye-diagram can be estimated by superposing the derived statistical output responses and expanding them to two-unit intervals (UIs). Accuracy and computational efficiency of the proposed method were validated. The proposed method enables extremely low BER analysis where conventional methods fail to estimate noises with low occurrence probabilities.

The proposed method was applied for SI/PI co-design and co-analysis of the high bandwidth memory (HBM) interposer channel. In the study, various design parameters affecting the SI/PI are considered. Specifically, impacts of the decoupling capacitor on the hierarchical PDN impedance and the statistical eye-diagram were focused on. From the derived statistical eye-diagrams of the HBM interposer channel, BER eye-diagrams and bathtub curves enabling timing and voltage analysis are derived. It is verified that the proposed method enables efficient SI/PI co-design and analysis by simultaneously plotting various results affecting the SI/PI of the target channel.

## Figures and Tables

**Table 1:** Occurrence probability equation summary when DBI coding is adopted.

	$P_{01\;(or\;10)-DBI}(N_{01},N_{10},N_{00},N_{11})$	$P_{00\;(or\;11)-DBI}(N_{01},N_{10},N_{00},N_{11})$
Case 1	If $N_{01}+N_{10}>N_{T}/2-1$ $P_{01-DBI}\left(N_{01},N_{10},N_{00},N_{11}\right)=0$	If $N_{01}+N_{10}>N_{T}/2$ $P_{01-DBI}\left(N_{01},N_{10},N_{00},N_{11}\right)=0$
Case 2	If $N_{01}+N_{10}=N_{T}/2-1$ $P_{01-DBI}\left(N_{01},N_{10},N_{00},N_{11}\right)\;$ $=P_{01}\left(N_{01},N_{10},N_{00},N_{11}\right)$	If $N_{01}+N_{10}=N_{T}/2$ $P_{01-DBI}\left(N_{01},N_{10},N_{00},N_{11}\right)=P_{01}\left(N_{01},N_{10},N_{00},N_{11}\right)$
Case 3 (Inversion)	If $N_{01}+N_{10}<N_{T}/2-1$ $P_{01-DBI}\left(N_{01},N_{10},N_{00},N_{11}\right)=P_{01}\left(N_{01},N_{10},N_{00},N_{11}\right)$+${\;P}_{00}\left(N_{00},N_{11},N_{01},N_{11}\right)$	If $N_{01}+N_{10}<N_{T}/2$ $P_{01-DBI}\left(N_{01},N_{10},N_{00},N_{11}\right)=P_{01}\left(N_{01},N_{10},N_{00},N_{11}\right)$+${\;P}_{00}\left(N_{00},N_{11},N_{01},N_{11}\right)$

**Table 2:** Accuracy and simulation time (estimation time for the proposed method) comparisons between HSPICE transient simulation and the proposed method.

	Transient Simulation ^1^ (@BER 10^−4^)		The Proposed Method ^2^	
	Eye-Height	Sim. Time	Eye-Height	Est. Time
1 Buffers, 1 Gb/s	1.108 V	15.81 s	1.138 V	3.52 s
4 Buffers, 1 Gb/s	1.063 V	78.32 s	1.034 V	6.75 s
4 Buffers, 2 Gb/s	1.045 V	80.11 s	1.011 V	8.10 s
4 Buffers, 8 Gb/s	0.619 V	3487 s	0.599 V	26.8 s
16 Buffers, 2 Gb/s	Fail to sim.		0.701 V	95.11 s

^1^ Workstation specification: Intel(R) Core(TM) i7-8700 CPU @ 3.20 GHz, 64.0 GB Installed memory (RAM). ^2^ Est. time: HSPICE step-responses simulation plus MATLAB calculation time.
